# Isolated Agenesis of the Corpus Callosum in a Four-Year-Old: A Case of Preserved Cognitive Function Despite Complete Corpus Callosum Absence

**DOI:** 10.7759/cureus.71104

**Published:** 2024-10-08

**Authors:** Mohammad N Almohammal, Saad Ali M Alqarni, Nasser Ali Alshahrani, Abdulrahman Ali M Algarni, Reef Turki M Alshahrani, Abdullah Saif Alqahtani

**Affiliations:** 1 Department of Pediatric Neurology, Maternity and Children Hospital, Bisha, SAU; 2 Department of Pediatrics, Abha Maternity and Children Hospital, Abha, SAU; 3 College of Medicine, University of Bisha, Bisha, SAU; 4 Department of Pediatrics, Maternity and Children Hospital, Bisha, SAU

**Keywords:** agenesis of the corpus callosum, cerebral malformation, intellectual impairment, neurocognitive dysfunction, neuropsychological tests

## Abstract

Agenesis of the corpus callosum (AgCC) is a rare congenital brain anomaly characterized by the partial or complete absence of the corpus callosum, a crucial structure responsible for interhemispheric communication. Neurological outcomes associated with AgCC vary widely, with presentation ranging from severe intellectual disabilities to normal cognitive function. The condition is often discovered incidentally due to the variability in its clinical presentation. This report discusses the case of a four-year-old Saudi boy with incidental AgCC identified following a minor head trauma.

The patient was born full-term through spontaneous vaginal delivery without perinatal complications. He presented with a history of mild head trauma leading to recurrent, non-progressive headaches localized in the occipital region. Despite the head trauma, there were no signs of increased intracranial pressure or other neurological deficits. A neurological examination revealed a head circumference above the 95^th^ percentile, but all other parameters, including cranial nerve function, motor strength, and reflexes, were within normal limits. Neuroimaging through computed tomography and magnetic resonance imaging unexpectedly revealed complete agenesis of the corpus callosum. Despite this structural anomaly, the patient’s cognitive function, assessed by the Mini-Mental State Examination and an IQ test, was within normal limits. The patient was managed conservatively for his head trauma and discharged with a recommendation for annual neurocognitive follow-up.

This case highlights the variable presentation of AgCC, where, despite the complete absence of the corpus callosum, the patient demonstrated no significant neurocognitive deficits. The findings align with existing literature suggesting that isolated AgCC can present with a broad spectrum of cognitive outcomes, from profound intellectual disability to near-normal function. The case underscores the importance of ongoing neuropsychological monitoring in individuals with AgCC, particularly as cognitive demands increase with age. Additionally, this case emphasizes the need for increased awareness and education among clinicians regarding the potential for late-emerging neurocognitive challenges in patients with AgCC.

## Introduction

Agenesis of the corpus callosum (AgCC) is an uncommon brain abnormality that may occur either on its own or in conjunction with other structural abnormalities as part of a complex congenital syndrome [1]. The defect is considered “complete” when there is a total absence of the corpus callosum (CC), or “partial” when only certain portions of the structure are absent. Although the physical effects of this defect are severe, the range of neurological symptoms found in those with AgCC vary from profound intellectual impairment to normal intelligence [2-4].

However, even in persons with isolated AgCC and no apparent neurological impairment, there may be modest neuropsychological changes when cognitive demands grow over time [5,6]. Therefore, it is necessary to conduct comprehensive and ongoing neuropsychological assessment of individuals with AgCC and a seemingly average intelligence quotient (IQ). Regrettably, due to the infrequency of AgCC, physicians have limited understanding of the neurological and cognitive characteristics of this abnormality. This lack of knowledge can result in delayed recognition of neurocognitive impairments and a postponed implementation of rehabilitative approaches designed to improve compensatory abilities in affected individuals. 

In this case report, we present an accidental discovery of isolated complete AgCC and grossly intact general intelligence in a four-year-old child, a condition characterized by the absence of the corpus callosum from the child’s brain. Despite the patient’s head trauma, this clinical case provides a valuable opportunity to enhance our understanding of AgCC as a rare condition.

## Case presentation

The patient was a four-year-old Saudi boy born of full-term spontaneous vaginal delivery with Apgar scores of 8 and 9 at one and five minutes after birth, respectively, no neonatal intensive care unit admission, and no perinatal complications. Medical examination showed no abnormal findings. The mother was free from any pre-existing medical disorders or abnormalities, had no past exposure to dangerous drugs before the pregnancy, and no family history of sudden death, chronic disease, or genetic disease.

The patient’s prior medical history was uneventful, with normal development and up-to-date vaccinations. The patient was born to parents who were first-degree relatives and had two siblings, all of whom were within the usual range of childhood development. His father had a condition called macrocephaly, which he believed was hereditary. 

The patient attended our center with a history of head trauma after he experienced a non-significant fall to the ground (i.e., less than his height). Since then, the patient developed recurrent headaches that were mild to moderate, on/off, non-progressing in severity or frequency, non-localizing but more at his occipital region, and not associated with any symptoms of high intracranial pressure such as vomiting, photophobia, diplopia, or squint. He had no history of fever, neck pain, or rigidity, but we noticed his head circumference was approximately 95th centile. He had no history of other central nervous system involvement, nor history of pain, loss of consciousness, abnormal movement, nystagmus, or speech abnormality. During his medical examination, the patient appeared conscious, alert, and oriented but with ptosis, weak facial expression, and mouth opening. His growth parameters were within the normal ranges apart from his head circumference being above 95th centile at 54 cm. Physical examination showed normal gait, cranial nerve reflexes, muscle strength, power tone, and deep tendon reflexes. Examination of the cerebella revealed no cerebellar dysfunction. Laboratory tests were performed, with a complete blood count showing electrolyte levels that were within normal ranges, as shown in Table 1. Liver function tests revealed values within normal ranges, as shown in Table 2.

**Table 1:** Complete blood count and electrolyte laboratory test results. * Normal laboratory values, Department of Pediatrics, Bisha Maternity and Children Hospital, Bisha, Kingdom of Saudi Arabia.

Complete blood count, Electrolyte	Lab values	Normal values*
Hemoglobin (HGB)	14.1 g/dL	12 – 15
WBC Count 10^3^/uL	5.6	4 – 10
Neutrophils count 10^3^/uL	2.1	2 – 7
Neutrophils %	38.9%	40 – 50
Lymphocytes %	55.4%	20 – 40
Eosinophils %	0.4%	1 – 6
Basophils %	0.5%	1 – 2
RBC 10^6^/uL	4.5	4.0 - 5.2
Platelet count 10^6^/uL	33	150 – 410
Potassium (K)	3.90 mmol/L	3.4 – 4.7
Calcium	9.4 mg/dL	8.8 – 10.8
Sodium (Na)	142.3 mmol/L	136 – 145

**Table 2:** Liver function test result. *Normal laboratory values, Department of Pediatrics, Bisha Maternity and Children Hospital, Bisha, Kingdom of Saudi Arabia. ALT: Alanine Aminotransferase, AST: Aspartate Aminotransferase

Normal values*	Lab values	Liver function test
0 – 50	0.33 U/L	ALT
5 – 43	20.5 U/L	AST
0.3 – 1.2	0.6 mg/dL	Total Bilirubin
0.0 – 0.50	0.40 mg/dL	Direct Bilirubin

**Figure 1:**
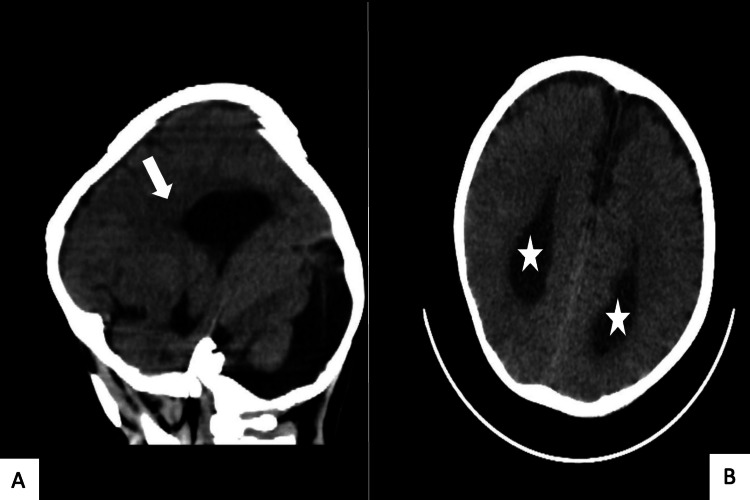
Brain computed tomography without contrast. Panel A: Suggestive of corpus callosum agenesis (white arrow). Panel B: Enlargement of lateral ventricular (white star).

The patient’s Mini-Mental State Examination (MMSE) results and IQ were within normal ranges. We sent him to the radiology department for brain computed tomography (CT) without contrast. As seen in Figure 1, the CT brain indicated no post-traumatic intracranial insult, acute lesion, or signs of hydrocephalus. However, the CT images were suggestive of AgCC, and the patient was sent to the radiology department for assessment via brain magnetic resonance imaging (MRI) without contrast.

**Figure 2:**
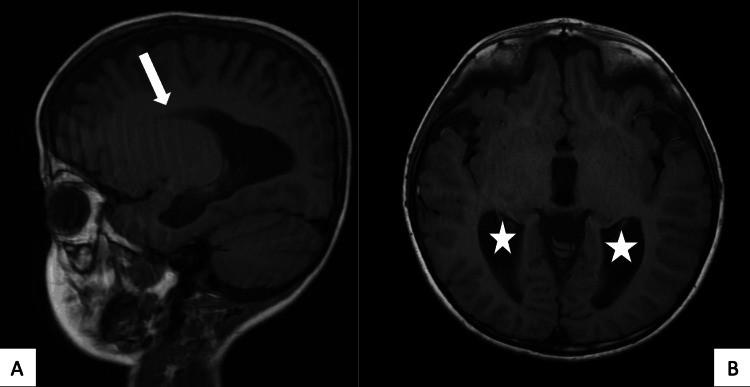
Brain magnetic resonance imaging. Panel A: Complete absence of corpus callosum (white arrow). Panel B: Dilated occipital horns and prominent trigones (white star).

As seen in Figure 2, MRI revealed complete absence of the corpus callosum with abnormal configuration of the lateral ventricles in the form of colpocephaly (dilated occipital horns and prominent trigones), narrow frontal horns with wide separation, parallel ordination of the racing car sign, hemispheric sulci reaching the high-riding third ventricle roof, and mildly dilated third and fourth ventricles. The patient finally received essential supportive treatment for his head trauma and was discharged with a scheduled annual medical follow-up for neurocognitive screening, The patient’s legal guardian gave written informed consent for the case to be published.

## Discussion

AgCC is a rare congenital abnormality of the brain where the corpus callosum is found to be partly or fully missing; its etiology remains unclear [7-9]. The condition usually manifests alone or alongside other brain anomalies; in cases where there are no other anomalies present, AgCC may be isolated [7]. Abnormal growth of the cerebellum, ventriculomegaly, and even other organ anomalies such as congenital heart disease are among the nervous system defects that may be associated with AgCC [8,9]. There are several risk factors associated with AgCC, including alcohol intake during pregnancy, intrauterine infection or injury, and genetic conditions, such as Dandy-Walker syndrome and Andermann syndrome [7]. It is important that AgCC patients receive regular checkups and neurological screening, as the condition has been shown to be a precursor to several neurological and cognitive disorders later in life [8]. In some cases, genetic factors, including chromosome abnormalities and syndromes such as Mowat-Wilson, have also been implicated in its development [9].

Education and training are also important to provide physicians with required knowledge of diagnosis and management of AgCC [7]. Early detection of AgCC and timely management of symptoms can help prevent neurological complications and improve patients’ quality of life. The standard method for diagnosing AgCC is through MRI or, in some cases, genetic testing. 

Herein, we report the case of isolated complete AgCC in a four-year-old patient with grossly intact general intelligence, providing intriguing insight into the variability of clinical manifestations associated with this congenital deformity. The absence of significant neurocognitive deficits in our patient, despite the complete lack of the corpus callosum, aligns with existing literature suggesting that isolated AgCC can present with a broad spectrum of cognitive outcomes, ranging from profound intellectual disability to near-normal function. A recent case study by Lamture et al. of a full-term newborn male child reported normal early neurological and cognitive development; similar to our case study, there were no early neurological anomalies, which reinforces the possibility of isolated cases of AgCC in the absence of these defects in spite of structural brain abnormalities [7]. The case study emphasized the importance of continuous follow-up, including regular assessments of developmental milestones, neurological examinations, and repeat MRI scans.

A case study by Guadarrama-Ortiz et al. of an eight-year-old Hispanic boy had similar findings to those of our case study, with normal neurological examinations, IQ score, and MMSE results [10]. Similar to our case study, they also found that the boy had no neuropsychiatric disorders or intellectual disabilities during early childhood. These similar findings suggest that AgCC can present with preserved cognitive function in early years. Therefore, long-term neurocognitive follow-up is important in managing the condition and preventing the development of neuropsychological deficits later in life. The potential role of genetic factors in AgCC should not be overlooked, as highlighted in both our case and the study by Guadarrama-Ortiz et al. [10]. 

Our case also exhibited some interesting similarities to a 2014 case study by Calabrò et al. of a 73-year-old woman with AgCC and frontotemporal dementia (FTD), as well as some differences. The case study highlights the variability in clinical outcomes associated with AgCC, as the elderly patient did not develop significant neuropsychiatric symptoms (e.g., memory impairment, dysexecutive syndrome, and behavioral abnormalities) until later in life [11]. Similarly, the young patient in our case study exhibited normal neurocognitive development and no significant deficits. The case study highlights the latent nature of AgCC, with the woman’s neuropsychiatric symptoms emerging only in late adulthood. As such, comorbid conditions such as FTD may influence the progression of AgCC later in life. In contrast, our case study showed that the young patient remained neurologically intact and functionally normal at a young age, despite the presence of AgCC. Although both cases emphasize the role of cerebral plasticity in mitigating the effects of AgCC, the elderly patient’s progressive cognitive decline suggests that age-related or comorbid neurodegenerative processes may eventually overcome these compensatory mechanisms. AgCC does not necessarily prevent normal development and functioning during childhood, but it may still lead to a variety of neuropsychiatric outcomes later in life, especially in the presence of comorbid conditions [12].

A study by Brown et al. suggested that AgCC patients may experience cognitive and social difficulties as they age, particularly when faced with complex, unfamiliar tasks that require higher-level integration between brain hemispheres [5]. This result is in line with existing evidence that AgCC does not cause a total “disconnection syndrome” but rather presents challenges that become more apparent as cognitive demands increase over time. Unlike the findings of Brown et al., our four-year-old patient currently does not exhibit the significant cognitive or motor deficits typically associated with AgCC, as his neuropsychological evaluation showed normal IQ and no observable impairments in motor coordination or processing speed. However, we note the key role that our patient’s young age played in his condition, and that the absence of current deficits in our patient does not preclude the possibility that these challenges may arise with age. The study by Brown et al. underscores the importance of ongoing neurocognitive monitoring to detect any emerging issues related to interhemispheric integration, processing speed, and complex cognitive tasks.

## Conclusions

It is paramount that medical professionals be aware of AgCC, albeit uncommon, and its potential complications, which may eventually cause irreversible disabilities. Although our case study presents a young patient whose brain’s current functioning appeared normal, the potential for later-emerging cognitive deficits, as highlighted by the literature, reinforces the need for vigilant long-term follow-up. Supported by existing case studies, our case study contributes to the understanding that AgCC can present with a wide spectrum of outcomes, which require personalized and adaptive care strategies throughout the patient’s life.
